# Anti-depression effectiveness of essential oil from the fruits of *Zanthoxylum bungeanum* maxim. on chronic unpredictable mild stress-induced depression behavior in mice

**DOI:** 10.3389/fphar.2022.999962

**Published:** 2022-09-20

**Authors:** Dandan Tang, Qi Liang, Mengmeng Zhang, Meiyan Li, Qing Zhang, Siyuan Zhang, Li Ai, Chunjie Wu

**Affiliations:** ^1^ Chengdu University of Traditional Chinese Medicine, Chengdu, China; ^2^ Sichuan College of Traditional Chinese Medicine, Mianyang, China; ^3^ School of Laboratory Medicine/Sichuan Provincial Engineering Laboratory for Prevention and Control Technology of Veterinary Drug Residue in Animal-Origin Food, Chengdu Medical College, Chengdu, China

**Keywords:** essential oil, the fruits of Zanthoxylum bungeanum maxim., CUMS, depression, HPA axis, PI3K/akt signaling pathway

## Abstract

The fruits of *Zanthoxylum bungeanum* Maxim. Was a popular traditional Chinese herbal medicine for pain relief, itching prevention, and diarrhea relief. The fruits of *Zanthoxylum bungeanum* Maxim. Essential oil (HEO) had an effect of improving anxiety and other emotional disorders. In this paper, we aim to systematically research the antidepressant effects of HEO on Chronic Mild Unpredictable Stimulation (CUMS) mice and explore the relevant molecular mechanisms. Experimental mice were exposed to CUMS for 8 weeks. Meanwhile, for 8 weeks, Sertraline hydrochloride (20 mg/kg/day) and HEO (50, 100, and 150 mg/kg/day) were administered by gavage. HEO treatment increased residence time of central zone in OFT and open-arm in EPM test but decreased immobility times in FST and TST. Moreover, HEO treatment improved the levels of 5-HT, DA, NE, and BDNF, but reduced CRF and CORT levels of the HPA axis in the hippocampus. Network pharmacology predicted the possible mechanisms for the antidepressant effects of HEO by regulation of PI3K/Akt signaling pathway. The mRNA expression of PI3K and Akt were increased, and immunofluorescence results in the hippocampus indicated that HEO treatment could increase the phosphorylation of PI3K and Akt. Besides, the viability of CORT-treated PC12 cells was significantly improved by HEO treatment. The AO-EB staining, MOMP analysis, and flow cytometry analysis results showed HEO inhibiting the CORT-induced apoptosis in PC12 cells significantly. Besides, the phosphorylation of PI3K and Akt in COTR-induced PC12 cells could increase by HEO treatment. In conclusion, HEO ameliorated depression behavior induced by CUMS, potentially via regulating HPA axis and activating PI3K/Akt signaling pathway to reduce neuronal apoptosis.

## Introduction

With the change of the social environment, the number of patients with depression is increasing. Depression has affected over 200 million people all over the world. The World Health Organization ranks depression as the third largest cause of the global disease burden and predicts that it will rank first by 2030 (James et al., 2018; Malhi and Mann 2018). Currently, oral antidepressants, such as fluoxetineare and venlafaxine, were the standard treatment for depression patients. However, these drugs have a long administration period and severe side effects (Anisman et al., 2008; DeFilippis et al., 2017). Consequently, discovering more reliable antidepressants with more minor side effects in the clinical treatment of depression was meaningful.

Traditional medicines derived from natural ingredients, especially essential oils of the plant, such as essential oil from the lavender (*Lavandula angustifolia*), *Citrus sinensis* (L.) Osbec, and bergamot (*Citrus bergamia* Risso.) have been widely used in mood disorders (López et al., 2017; Zhang L et al., 2019; Yang et al., 2021; Peng et al., 2022). In cooking, the fruits of *Z. bungeanum* Maxim. a popular spice with distinct flavors and aromas, were used. Furthermore, the fruits of *Z. bungeanum* Maxim. Was a popular traditional Chinese herbal medicine for pain relief, itching prevention, and diarrhea relief. The fruits of *Z. bungeanum* Maxim. mainly contains essential oils, alkaloids, amides and flavonoids, and other chemical components (Zhang et al., 2017). Among these components, the essential oil was one of the active sites of the fruits of *Z. bungeanum* Maxim. Essential oil containing d-limonene, Linalool, and linalyl acetate et al. Yang (2008). The fruits of *Z. bungeanum* Maxim. essential oil (HEO) has anti-tumor, anti-inflammation, antipruritic, and other pharmacological activities (Li et al., 2016; Hou et al., 2021; Zhou et al., 2022). Recently, with the development of medicinal values for the fruits of *Z. bungeanum* Maxim. Wei et al. demonstrated that intragastric administration HEO (100 mg/kg and 200 mg/kg) could improve mood of anxious rats. Its primary mechanism could be linked to the regulation of the HPA axis and gut microbiota (Wei et al., 2021). Whether HEO has an ameliorative effect on depression-like mice remains uninvolved.

In the present study, we investigate whether the HEO could ameliorate depression behavior induced by CUMS. In addition, the mechanism of HEO improving the CUMS-treated mice was predicted by network pharmacology, and preliminarily verified in CORT-treated PC12 cells.

## Materials and methods

### Acquisition of medicinal materials and preparation of essential oils

The fruits of *Zanthoxylum bungeanum* Maxim. were obtained from Maoxian (Sichuan, China). The obtained samples were kept in the laboratory (No. HJ20210712). The fruits of *Zanthoxylum bungeanum* Maxim. was dried at 50°C. Then it was crushed and passed through a 50 mesh sieve. HEO was obtained by steam distillation for 5–6 h. The yield of HEO was 4.98% (v/w) and the density was 0.8027 g/ml.

### GC-MS analysis of HEO

GC-MS was used to examine the constituents of HEO. HP-5MS capillary column (30 m × 0.25 mm, 0.25 μm) was used for chromatography. The temperature rise is as follows: the initial temperature was 50°C, lasting for 2 min, and 5°C/min increases to 150°C; 200°C for 3 min; The temperature was then raised to 240°C at a rate of 25°C/min. The flow rate was 1 ml/min, the split ratio was 3:1, and the injection volume was 1.0 μl. The ion energy of electron impact ionization was 70 ev, and the mass range of scanning was 50–600. The collected compound fragment information was used to identify compounds using the NIST mass spectrometry database (Washington, United States). The relative content of each chemical component of HEO were expressed as the percentage of its peak area to the total peak area.

### Animals

ICR mice (male, 18–20 g) were obtained from Beijing Sibeifu experimental animal Co., Ltd. Mice were housed in a single cage (except for the control group). Before the start of formal experiment, mice were adaptively fed for 7 days. All animal experimental procedures were carried out in accordance with the guide for the care and use of laboratory animals issued by the National Institutes of health and approved by the animal protection and ethics committee of Chengdu University of traditional Chinese medicine.

### Experimental design

32 mice were grouped into four groups randomly (*n* = 8) in the depression behavioral (DB) test: control, HEO (50 mg/kg), HEO (100 mg/kg), and HEO (150 mg/kg). Our preliminary experiment determined the dose of HEO in this study. Experimental mice of different doses of HEO were administered by an intragastric gavage for 14 days, and 1% CMC-Na was used to suspend the HEO. Meanwhile, mice of control groups mice were administered only with 1% CMC-Na (10 ml/kg). On the 13th and 14th day, the FST and TST trials were performed 1 h after the HEO treaatment. Figure 2A shown the experimental schematic diagram.

In the CUMS test, mice were grouped into six groups randomly (*n* = 8): control, model, Sertraline hydrochloride (20 mg/kg) treated group (SERT group), and three HEO treatment groups with doses of 50, 100, and 150 mg/kg/day, respectively. SERT group, HEO groups were given oral administration of drugs. In contrast, all the groups were still randomly exposed to one of the stimuli daily. 1% CMC-Na (10 ml/kg/day) was given to the control and model groups by gavage. After completing the behavioral test, the mice were sacrificed. Whole blood and brain tissue samples were rapidly obtained. The blood sample was centrifuged (10 min, 3000 rpm), and the supernatant was collected as serum. *Hippocampus* was dissected on ice. Both serum and hippocampus tissues were stored at −80°C. Besides, some of brain samples fixed in 4% paraformaldehyde were used for H&E, Nissl staining, and immunohistochemical assay. Figure 3A exhibited the experimental schematic diagram.

### The program of chronic unpredictable mild stress procedure (CUMS)

Refer to relevant literature and adjust the types of chronic mild and unpredictable stimuli (Ma, et al., 2021). After 7 days of adaptive feeding, mice in the CUMS group, SERT group and three HEO groups were housed in a single cage and exposed to the following mild stimulation environment for 8 weeks: wet bedding for 24 h; Restraint for 1 h (the restraint container was made of 50 ml EP tube, and the restraint device only restricts the movement of mice, and does not restrict the free breathing and air circulation of mice); Food and water deprivation for 24 h (19:00–19:00); ice water swimming at 0°C, in a cylindrical tank filled with water (30 cm × 15 cm × 10 cm) for 10 min; clamp tail for 5 min; turning night into day; inclined cage for 24 h. All stimuli were performed at different times in random order, and the different stimulus was given for two consecutive days.

### FST and TST trails

Mice were individually placed in a plastic cylinder (30 cm × 18 cm) with 20 cm water (25°C). The mice were forced to swim during a period of 6 min. The first 2 min was the adaptation time, and only the last 4 min was recorded. The defined immobility time was that a mouse floats in the water without struggling and only performs the actions required to expose its head to the water surface (Ma et al., 2021). The obtained data were analyzed and processed by RWD smart 3.0 software (Shenzhen, China).

The tail suspension test was conducted with reference to relevant literature (Zhang K et al., 2020). That is, the tail of each mouse was suspended for 6 min with tape. The first 2 min was the adaptation time, and the immobility time of the last 4 min was recorded. The obtained data were analyzed and processed by RWD smart 3.0 software (Shenzhen, China).

### SPT trails

In SPT, the mice were housed in a single cage. The first 24 h was the sugar water adaptation stage. The mice were allowed to drink two bottles of sucrose solution (1%, m/v). After the adaptation in the sugar water phase was completed, a new bottle of sucrose solution and distilled water were supplied to the mice in the second 24 h. In the third 24 h, mice were deprived of food and water. In the fourth 24 h, provide 100 g sucrose solution and 100 g pure water solution, and exchange the position of the water bottle every 6 h. The preference rate of mice for sucrose solution was calculated (Li W et al., 2020).

### OFT trails

The device for OFT was provided by RWD Life Sciences Co., Ltd. The open field’s bottom was divided into a peripheral zone and a central zone (20 × 20 cm). Mice were left alone in the center of the central area for 6 min to explore. The time spent in the central zone during the final 5 min was recorded. To eliminate residual odors, the OFT device was cleaned with 75% ethanol between tests (Xue et al., 2016).

### The elevated plus-maze test

Mice were placed in the experimental environment for 30 min. The elevated cross labyrinth device was provided by RWD Life Sciences Co., Ltd. Mice were placed on a central platform facing one of the open arms at the start of the EPM test and then allowed to explore freely for 5 min. An automatic video tracking system recorded the retention time of mice in open and closed arms (smart basic, China). The shorter retention time in the open arm, the higher the anxiety state. The device must be cleaned between the two tests to remove any residual odor (Wang et al., 2021).

### ELISA

ELISA kit (Ruixin Co., Ltd., China) were used to determine the contents of 5-HT, DA, NE, CRF, CORT, and Brain derived neurotrophic factor (BDNF) in the hippocampus. *Hippocampus* samples were homogenized at 4°C and then centrifuged before determination, and the supernatant was taken for content determination. The test process was done following the instructions of the kit strictly.

### Histological analysis

Brain tissues were collected and fixed for 48 h in 4% paraformaldehyde before ethanol dehydration, paraffin embedding, and 5 μm slicing. Both H&E and Nissl staining were performed (COSSIM FR-4A, China).

### Database construction

Use the compounds identified under “2.1”to download the structure in PubChem. The Swiss Target Prediction database (http://www.swisstargetprediction.ch/) was used to find HEO compound target proteins. The species was named“*Homo sapiens*”, the corresponding target genes of HEO compounds were collected. These targets were combined with limited to “probability>0”, and the duplicate genes corresponding to the compounds are deleted. Gene Cards (https://www.genecards.org/) was used to obtain depression-related targets. Finally, the intersection genes of HEO and depression were obtained via the Venny online website (https://bioinfogp. cnb. csic.ES/tools/Venny/index.html).

### Network construction

Cytoscape software was adopted to display network, analyze, edit data, and visualize it. Furthermore, the network analyzer plug-in was used to calculate the number of nodes, K-degree, edge number, and other important parameters in order to evaluate the topological characteristics of each node in the network, clarify the more important components and goals of HEO, and further explain its molecular mechanism for treating depression in a scientific and reasonable manner.

### PPI network construction

The intersection genes obtained through “Database Construction” were used to build a protein-protein interaction (PPI) network with a “*Homo sapiens*”species restriction. PPI networks with scores greater than 0.7 were kept. To visualize the PPI network, the analysis results were downloaded in CSV format and imported into Cytoscape (version 3.9.0). The cytohubba plug-in software was also used to analyze and select central targets in the PPI network.

### Go functional annotation and KEGG pathway analysis

R language software was used to the enrichment analysis to perform the biological terminology classification and pathways of the screened target proteins. We select relevant functions or pathways under *p* < 0.05, and only the top 20 information will be displayed in the results.

### RT-qPCR assay

Animal RNA Extraction Kit (Foregene, China) was adopted to obtain RNA from hippocampus tissue samples. RNA concentration was determined by a nanophotometer (NP80, IMPLEN, Germany). To ensure the purity of RNA, the optical density ratio (OD260/OD280) at 260 and 280 nm was controlled within the range of 1.8–2.1. Then, cDNA was obtained by reversing 1 μg RNA. After adding premier, SYBR Green (Foregene, China) and cDNA mixture, RT-qPCR was carried out using ABI step one plus analyzer (Applied Biosystems, USA) under the conditions followed as: 95°C for 3 min, then 40 cycles at 95°C for 10 s and 60°C for 30 s. RNA primers of GAPDH, BDNF, PI3K and Akt were designed and synthesized by Qingke biotechnology company (Beijing, China). Primers were shown in Table 1. 2^−ΔΔCT^ was performed to calculate the mRNA levels of BDNF, PI3K, and Akt.

**TABLE 1:** Primer sequences used for RT-qPCR analysis.

Gene name	Sequence (5′-3′)	The length of primer (BP)	Tm/°c
GAPDH	F: CAG​TGG​CAA​AGT​GGA​GAT​TGT​TG	23	57.77
	R:TCGCTCCTGGGAAGATGGTGAT	21	57.57
BDNF	F:TCCGGGTTGGTATACTGGGTT	21	57.57
	R: GCC​TTG​TCC​GTG​GAC​GTT​T	19	57.32
PI3K	F:GGCACAGACTTGGTGTTTT	19	53.01
	R:TCCCCCAGTACCATTCAGC	18	54.90
Akt	F:TGCACAAACGAGGGGAA	17	52.19
	R:CGCTGATCCACATCCTGA	18	54.90

### Immunohistochemistry

Immunohistochemical fluorescence staining was performed to detect protein expression. Brain tissue samples were fixed (4% paraformaldehyde) and cut into 5 μm sections. Then, the tissue sections were stained with anti-p-PI3K, PI3K, p-Akt and Akt antibodies (dilution ratio of p-PI3K/p-Akt was 1:200, PI3K and Akt was 1:400, Ambrt, China). After washing with PBS, the fluorescent secondary antibody (anti rabbit/anti mouse) were incubated for 1 h in the dark at room temperature before being washed off, and the anti-quencher containing DAPI was added. The expression of proteins in the hippocampus was photographed by digital microscope (COSSIM, China). Fluorescence intensity was analyzed by ImageJ software (Media Cybernetics, USA).

1×10^5^/ml PC12 cells were seeded in glass dishes and incubated for 24 h at 37°C and 5% CO_2_. PC 12 cells were fixed at -20°C for 20 min (4% paraformaldehyde). Subsequently, PC12 cells were stained with primary antibodies against PI3K/p-PI3K and Akt/p-Akt and fluorescent pigment conjugated secondary antibody. An anti-fluorescence quenching agent containing DAPI was added. Then a confocal microscope (Leica, Germany) was used to take photographs under a ×40 objective.

### Cell culture

PC12 cells were grown in DMEM complete medium with 10% FBS (v/v), 1% double antibody. The temperature was 37°C, and the concentration of CO_2_ was 5%. PC12 cells were treated with HEO (25, 50, and 100 μg/ml) for 4 h before being treated with 500 μM CORT for 24 h. The model group received the same amount of complete medium and then received CORT.

### Determination of cell viability

25 μg/ml, 50 μg/ml, and 100 μg/ml HEO were applied to PC12 cells for 4 h before being treated with 500 μM CORT for 24 h. The cells in the model group were treated with an equal amount of DMEM before being injured with CORT. The model group cells were treated with equal amount of DMEM and then injured with CORT. Following the experiment, aspirate the medium and add 10 μL of CCK-8 to each well. The absorbance value was detected by the microplate reader (Bio-Rad, Hercules, CA, USA) after 30 min of incubation, and the cell survival rate was calculated using the absorbance value.

### AO/EB staining and assessment of MMP

PC12 cells were seeded into 6-well plates and treated according to the instructions in “Cell Culture”. The cells were then stained with an AO/EB staining solution (1:1, V/V) and incubated in the dark for 30 min. The dye was then washed away and photographed using a laser confocal microscope. The MMP was measured using the JC-1 probe. The treated PC12 cells were incubated with the JC-1 probe for 15 min in the dark at 37°C. The photographed using a laser confocal microscope software (Leica, SP8 SR, Germany). The ratio of green fluorescence and red fluorescence in each group was compared and analyzed.

### Apoptotic rate measured by flow cytometer

The apoptosis of PC12 cells induced by CORT was detected using flow cytometry analysis. PC12 cells were cultured into six well plates with HEO (25, 50, 100 μg/ml) and the presences of CORT (500 μM) for 24 h. Cells were stained using Annexin V-FITC/PI kit, and finally the flow cytometry analysis was used to determine the apoptosis rate of the samples (BD, New York, USA).

### Statistical analysis

*T* Test in GraphPad Prism nine software (La Jolla, USA) was adopted to perform the statistical analyses between two groups. The significant level was considered as *p* < 0.05.

## Results

### Chemical constituents analysis of the HEO

**FIGURE 1:**
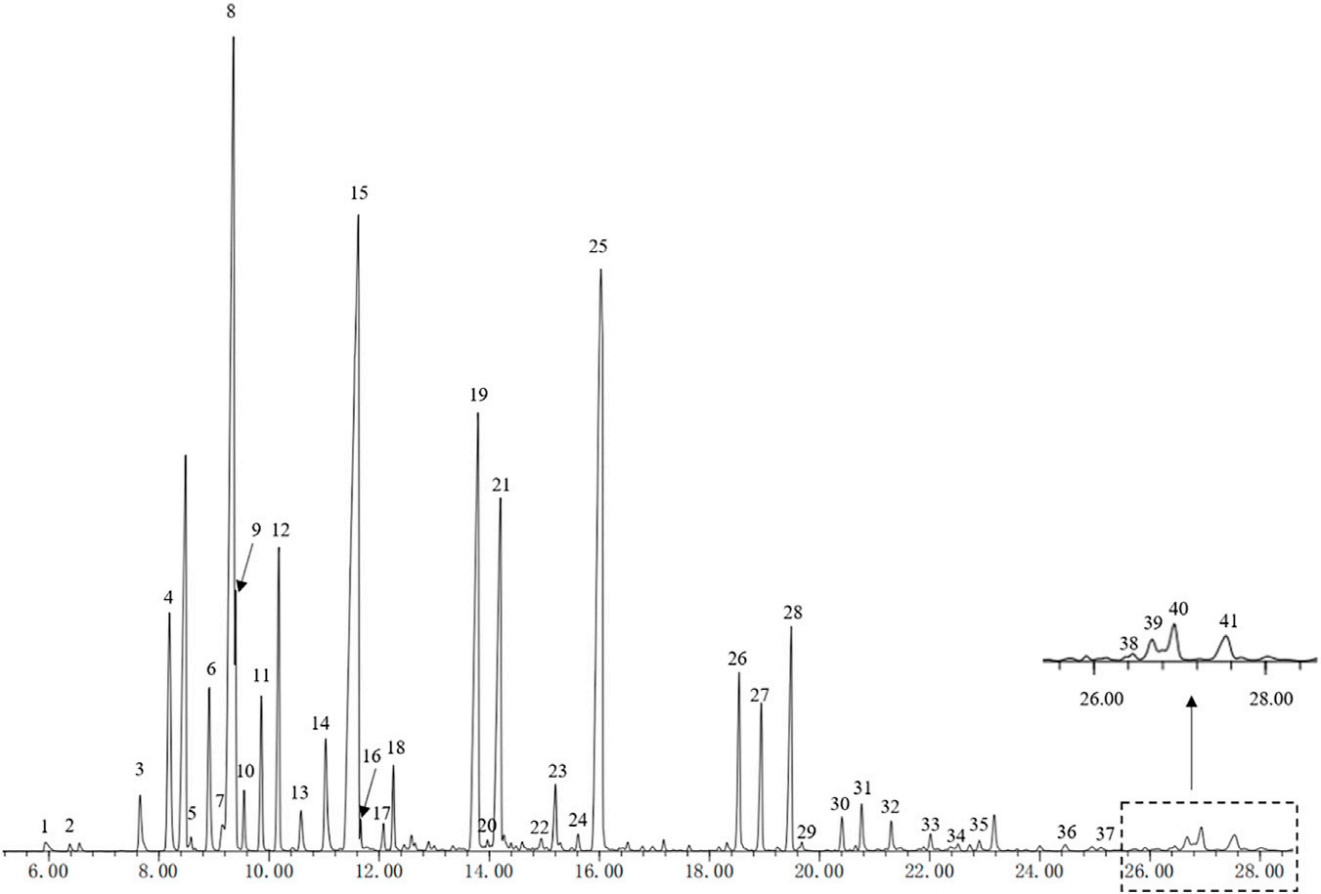
GC-MS detected 41 peaks (Relative Content>0.1%) of HEO. HEO, the essential oil from the fruits of *Z. bungeanum* Maxim.; GC-MS, Gas chromatography-mass spectrometry.

The chemical components of HEO were detected by GC-MS analysis. In the present study, the information of the compounds were matched reports in the literature and GC mass spectrometry data. 41 compounds (relative content>0.1%) were identified from the total ion chromatogram (28.6 min) of HEO (Figure 1). These compounds were subsequently identified and the Peak area normalization method was adopted to calculate their relative contents. Among the 41 identified compounds, d-Limonene (peak 8, 15.17%), Linalool (peak 15, 19.25%), and Linalyl acetate (peak 25, 13.85%) were the main compounds. Of these 41 compounds, 14 with a relative content of more than 1% and other compounds information of HEO were shown in Supplementary Table S1.

### Effects of HEO on mice with DB model

**FIGURE 2:**
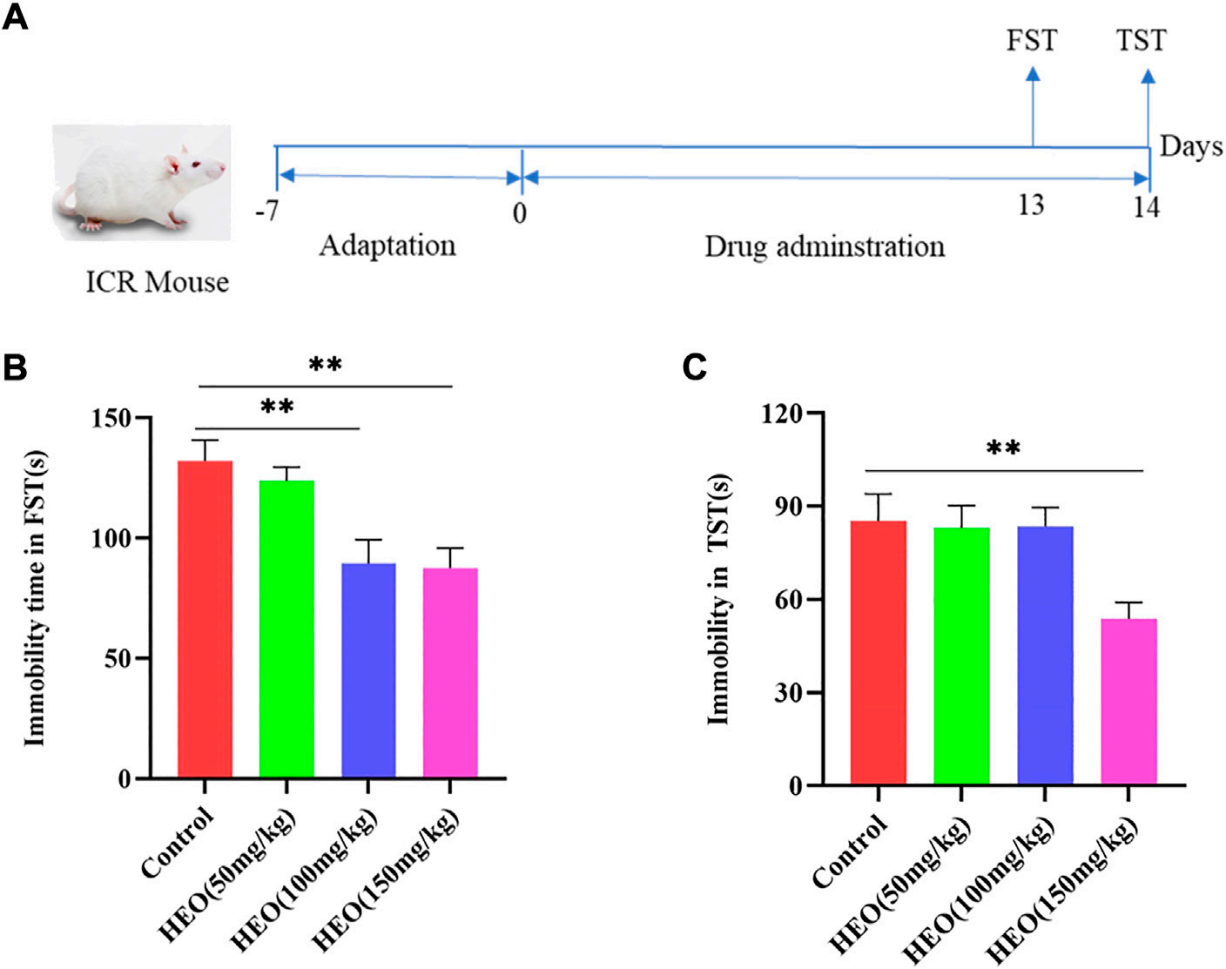
HEO treatment decreased the immobility times in FST and TST. The experimental schematic diagram **(A)**; immobility time in FST **(B)** and immobility time in TST**(C)**. Data were expressed as the mean ± SEM (*n* = 8). **p < 0.01 compared with the control group. HEO, the essential oil from the fruits of *Z. bungeanum* Maxim.; FST, forced swimming test; TST, tail suspension test.

DB model was used for the preliminary screening for antidepressant activity of drugs. In the DB model test, HEO administration (50, 100 and 150 mg/kg) group can reduce immobility time in FST and TST. The control group’s immobility time in the FST was 132s, while the immobility times of the 50 mg/kg, 100 mg/kg, and 150 mg/kg groups were 123.6, 89.5, and 87.5s, respectively. The mice immobility time was reduced by 50 mg/kg HEO, but there was no statistical difference; however, the 100 mg/kg and 150 mg/kg groups had a significant difference (*p* > 0.05, *p* < 0.01, *p* < 0.01 vs control group, Figure 2B). In TST, treatment groups of 50, 100, and 150 mg/kg could also reduce immobility time, but only the HEO 150 mg/kg group had a significant statistical difference (*p* > 0.05, *p* > 0.05, *p* < 0.01, respectively, Figure 2C).

### HEO affection on attenuating of depression behavior in CUMS treatment mice

**FIGURE 3:**
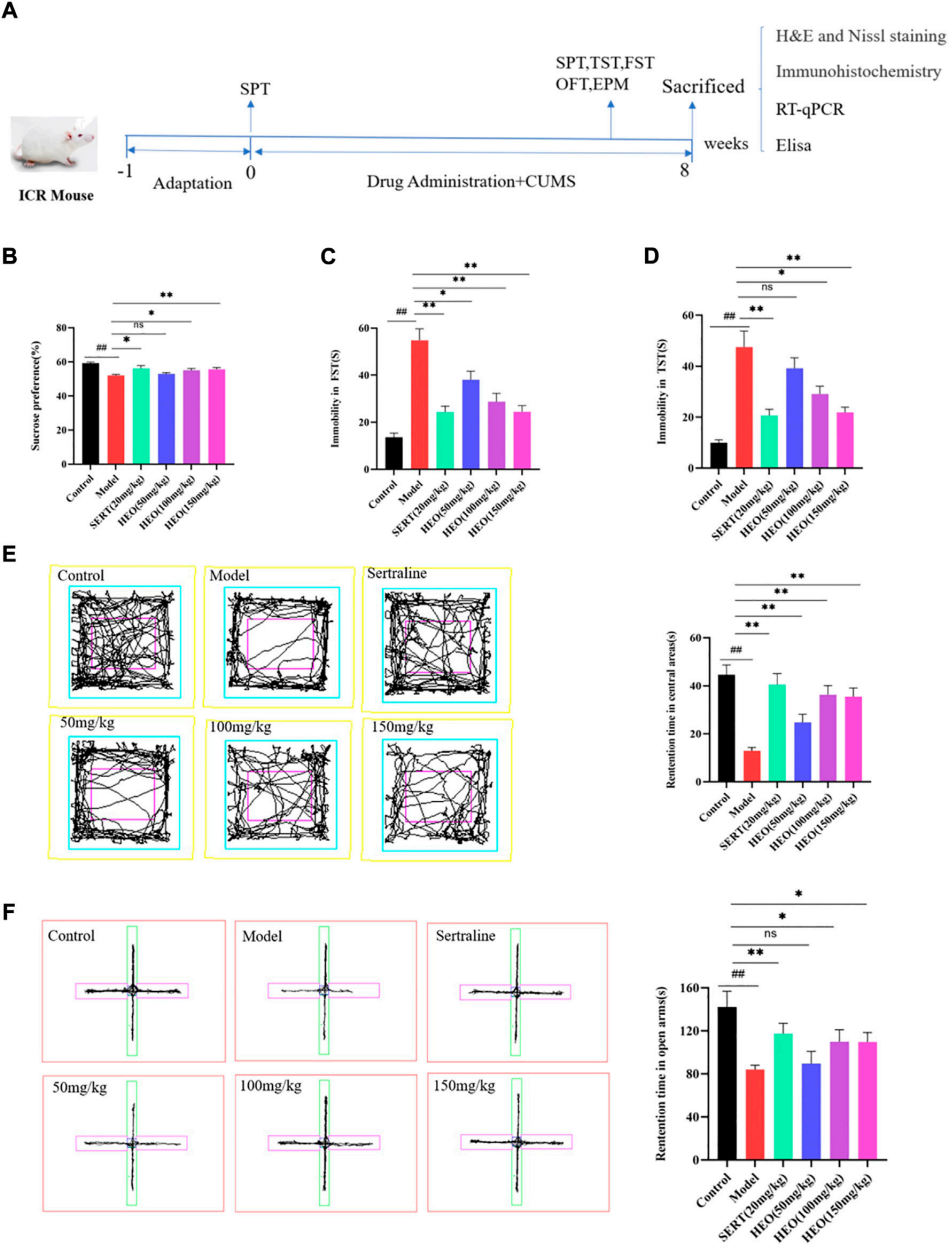
The Effect of HEO on depressive-like behavior treated by CUMS. The experimental schematic diagram **(A)**; Sucrose preference test **(B)**; Forced swimming test **(C)**; Tail suspension test **(D)**; Open field test **(E)**; The elevated plus-maze (Note:The rose red one represent open-arms and the green one represent close-arms) **(F)**. Data were expressed as mean ± SEM (*n* = 8). ^##^ *p* < 0.01 vs control group; *p < 0.05,**p < 0.01 vs the CUMS group. HEO, the essential oil from the fruits of *Z. bungeanum* Maxim.; SERT, Sertraline; CUMS, chronic unpredictable mild stress.

To further verify whether HEO has antidepressant activity and potential mechanisms, a CUMS induced depression-like mice was used for subsequent studies. During CUMS treatment, different doses of HEO and SERT were given by gavage at the same time. As shown in Figure 3B, the mice in the CUMS group reduced the sucrose preference rate to 52.0%, which was statistically significant when compared to the control group (*p* < 0.01). SERT and HEO treatment, on the other hand, increased sucrose preference. The sucrose preference increased to 56.1%, 52.9, 55.0, and 55.6% in the SERT Group and HEO (50, 100, and 150 mg/kg) groups, respectively (*p* < 0.01, *p* > 0.05, *p* < 0.05, *p* < 0.01). Second, the CUMS group’s FST immobility time was significantly increased (54.8 and 13.6s, respectively, *p* < 0.01 vs control group). SERT group (*p* < 0.01) and HEO (50, 100 and 150 mg/kg) significantly reduced mouse immobility time (*p* < 0.05, *p* < 0.01, and *p* < 0.01) (Figure 3C). The immobility time of CUMS-treated mice in TST was significantly increased when compared to the control group (*p* < 0.01). SERT (*p* < 0.01) and HEO (50, 100, and 150 mg/kg) groups reduced immobility time after treatment (*p* > 0.05, *p* < 0.05, *p* < 0.01), with the HEO (150 mg/kg) group demonstrating a strong antidepressant effect (Figure 3D). CUMS treatment significantly reduced residence time in the central area compared to the control group in the OFT test (*p* < 0.01). HEO (50, 100, and 150 mg/kg, respectively, *p* < 0.01, *p* < 0.01, *p* < 0.01) or SERT treatment (*p* < 0.01), improved this reduction (Figure 3E). The mice after the intervention of HEO and SERT showed higher autonomous activity ability, and HEO had a tendency to improve the “loss of interest”of mice. In EPM test, the rentention time in open arm of CUMS treatment group was 81.92s, which was significantly reduction compared with control group of 142.19s (*p* < 0.01), While HEO (50, 100, and 150 mg/kg, *p* > 0.05, *p* > 0.05, and *p* > 0.05, respectively) or SERT treatment (*p* < 0.01) significantly increased mouse residence time in the open arm (Figure 3F). The results above indicate that HEO could effectively improve the related behaviors of CUMS depression like mice.

### HEO effective on neurotransmitters and HPA axis in CUMS-treated mice

**FIGURE 4:**
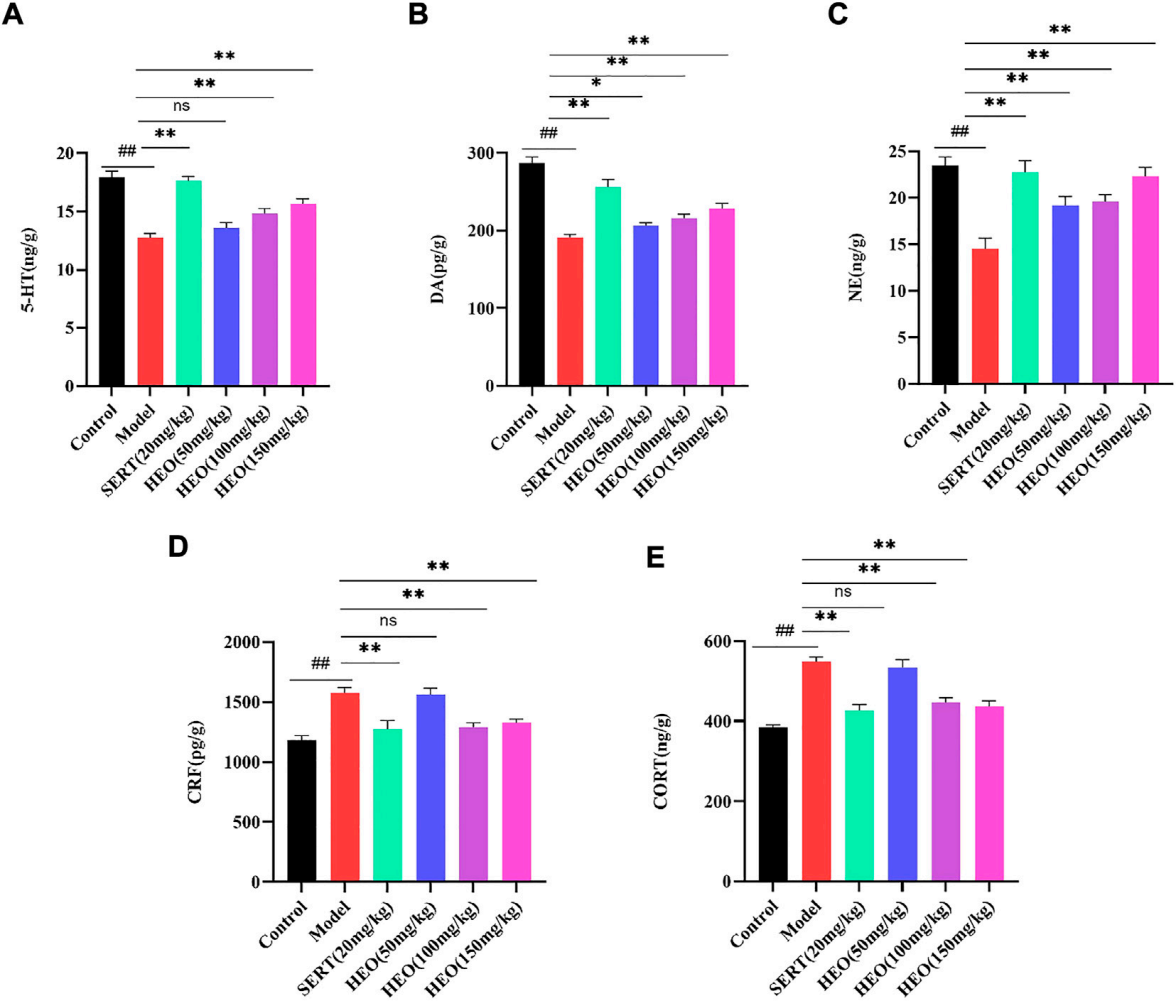
Effects of HEO on brain monoamine neurotransmitters in *Hippocampus* and HPA axis hormone levels. 5-HT**(A)**; DA**(B)**; NE level **(C)**; CRF level **(D)**; CORT level **(E)**. Data were expressed as mean ± SEM (*n* = 8). ^##^ *p* < 0.01 vs control group; *p < 0.05 and **p < 0.01vs model group. HEO, the essential oil from the fruits of *Z. bungeanum* Maxim; 5-HT, serotonin; DA, dopamine; NE, norepinephrine; CRF, corticotropin-releasing factor; CORT, corticosterone.

To further understand HEO’s antidepressant mechanism, we investigated its effects on neurotransmitters and hormone levels of the HPA axis in the hippocampus. As shown in Figures 4A–C, the levels of 5-HT, DA, and NE in the hippocampus of CUMS-induced mice were significantly decreased (*p* < 0.01, *p* < 0.01, and *p* < 0.01 vs control group). Nonetheless, administration of HEO (50, 100, and 150 mg/kg) in the hippocampus improved these reduction (5-HT: *p* > 0.05, *p* < 0.01, and *p* < 0.01, respectively; DA: *p* < 0.01, *p* < 0.01 and *p* < 0.01, respectively; NE: *p* < 0.05, *p* < 0.05 and *p* < 0.01, respectively). After SERT treatment, monoamine neurotransmitters showed the same trend (5-HT: *p* < 0.01, DA: *p* < 0.01, NE: *p* < 0.01). Furthermore, mice exposed to CUMS procedures had higher levels of CRF and CORT in the hippocampus (*p* < 0.01, *p* < 0.01vs control group, respectively) (Figures 4D,E). HEO (100 and 150 mg/kg) treatment significantly reduced CRF and CORT levels in the hippocampus (*p* < 0.01, *p* < 0.01, respectively). This trend was also seen following SERT treatment (CRF: *p* < 0.01; CORT: *p* < 0.01).

### HEO possessed potential protective effects against CUMS-treated mice

**FIGURE 5:**
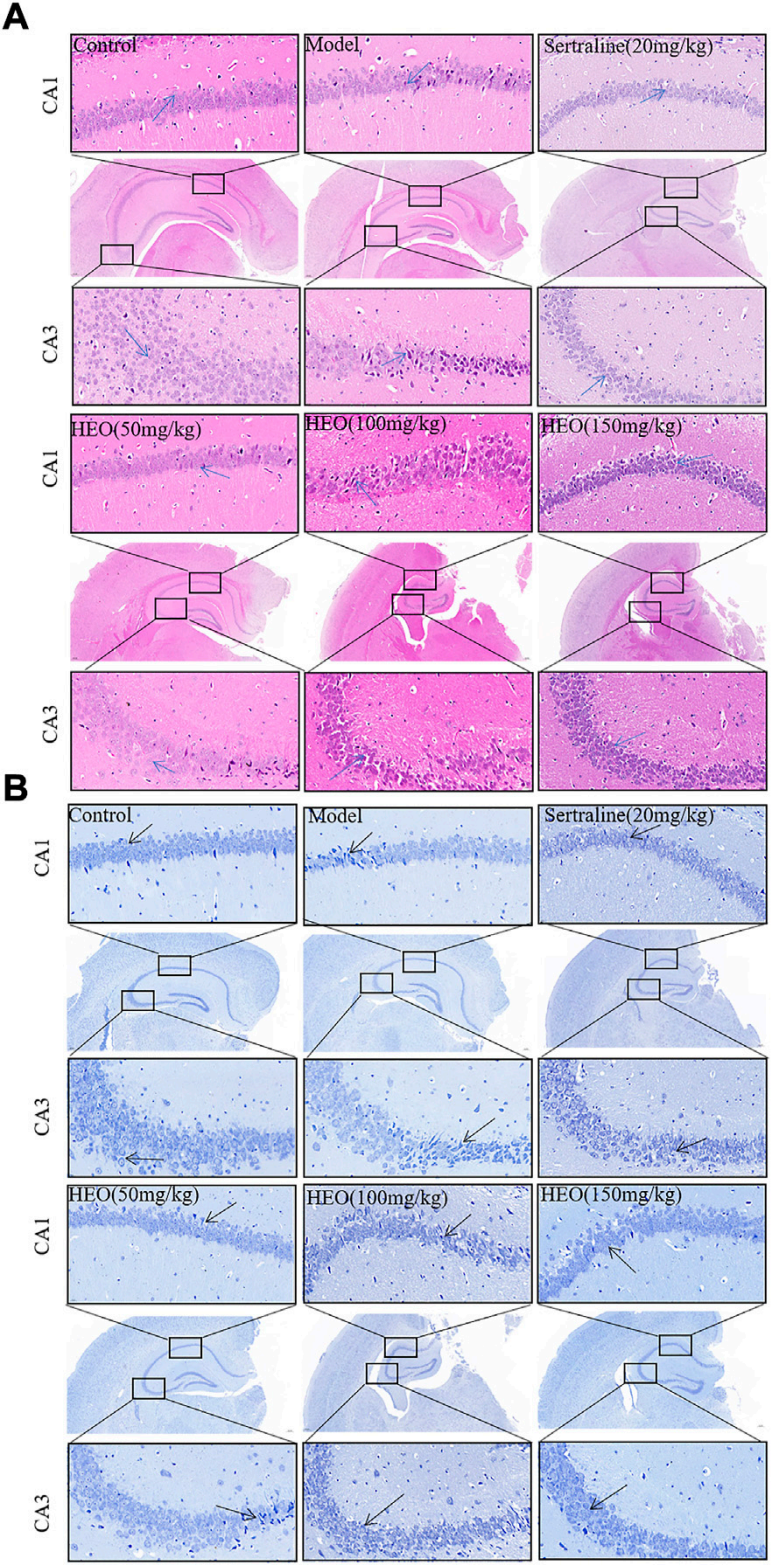
Histological analysis and Nissl staining analysis of hippocampal CA1 and CA3 areas (40×and 400×): H&E staining **(A)**; Nissl staining **(B)**.

As shown in Figure 5, results of the H&E (5A) and Nissl staining (5B) were presented. H&E staining results exhibited that structure of neurons in hippocampal CA1 and CA3 areas were completely, and no pathological changes were observed. In addition, the neuron’s outline in the control group was clear, and the shapes were large and round (arrow). However, in model group, neurons were shrinkage, volume reduced, polygonal basophilic changes, nuclei unclear, and cytoplasm decreased and widened intercellular spaces in hippocampal CA1 and CA3 areas. The results of the H&E staining showed that, when compared to CUMS treatment group, the pathological changes in the hippocampus could be improved after stomach irrigation with SERT (20 mg/kg) and HEO (50, 100, and 150 mg/kg) (Figure 5A). Furthermore, results of Nissl staining were summarized in Figure 5B. Neurons in the hippocampus were damaged in the model group, with irregular and loose distribution, cytoplasm pyknosis, and Nissl body disintegration. In contrast, HEO (50, 100, and 150 mg/kg) and SERT (20 mg/kg) could increase the number of Nissl bodies compared to the CUMS treatment mice. The above results indicate that HEO has a potential protective effects on CUMS induced hippocampal neuron injury in mice.

### HEO effective on BDNF in CUMS-treated mice

**FIGURE 6:**
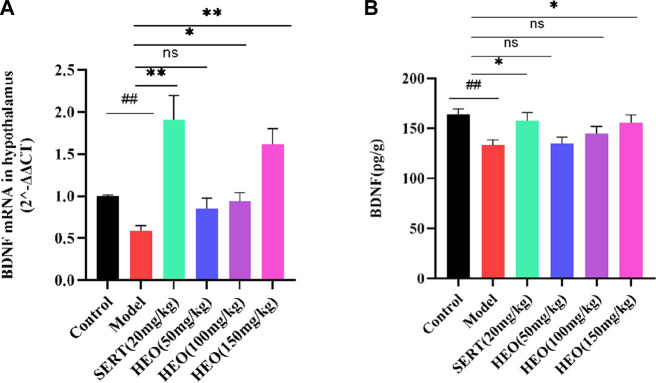
Effects of HEO on BDNF mRNA **(A)** and protein **(B)** expressions in the hippocampus. Data were expressed as mean ± SEM (*n* = 6). ^##^ *p* < 0.01 vs control group; *p < 0.05, **p < 0.01 vs the CUMS group. BDNF, brain-derived neurotrophic factor.

More and more evidence has come out that stress might negatively regulate the BDNF. Next, we adopted RT-qPCR to test whether HEO could increase the mRNA of BDNF in the hippocampus. Compared to control group, CUMS treatment group significantly decreased the mRNA expression of BDNF (*p* < 0.01). In contrast, CUMS-induced mRNA expression in the hippocampus was significantly reversed by 100 and 150 mg/kg HEO treatment (*p* < 0.05, *p* < 0.01, respectively). However, when compared to the CUMS-treated group. And the level of BDNF increased significantly (*p* < 0.01) after sertraline treatment (Figure 6A). Furthermore, the protein expression of BDNF was measured by Elisa kit. The CUMS treatment group significantly reduced BDNF protein expression (*p* < 0.01 vs control group). In contrast, sertraline and 150 mg/kg HEO treatment significantly reversed the CUMS-induced reduction in BNDF levels in the hippocampus (*p* < 0.05, *p* < 0.05, respectively) (Figure 6B).

### PPI analysis and target biological function analysis

**FIGURE 7:**
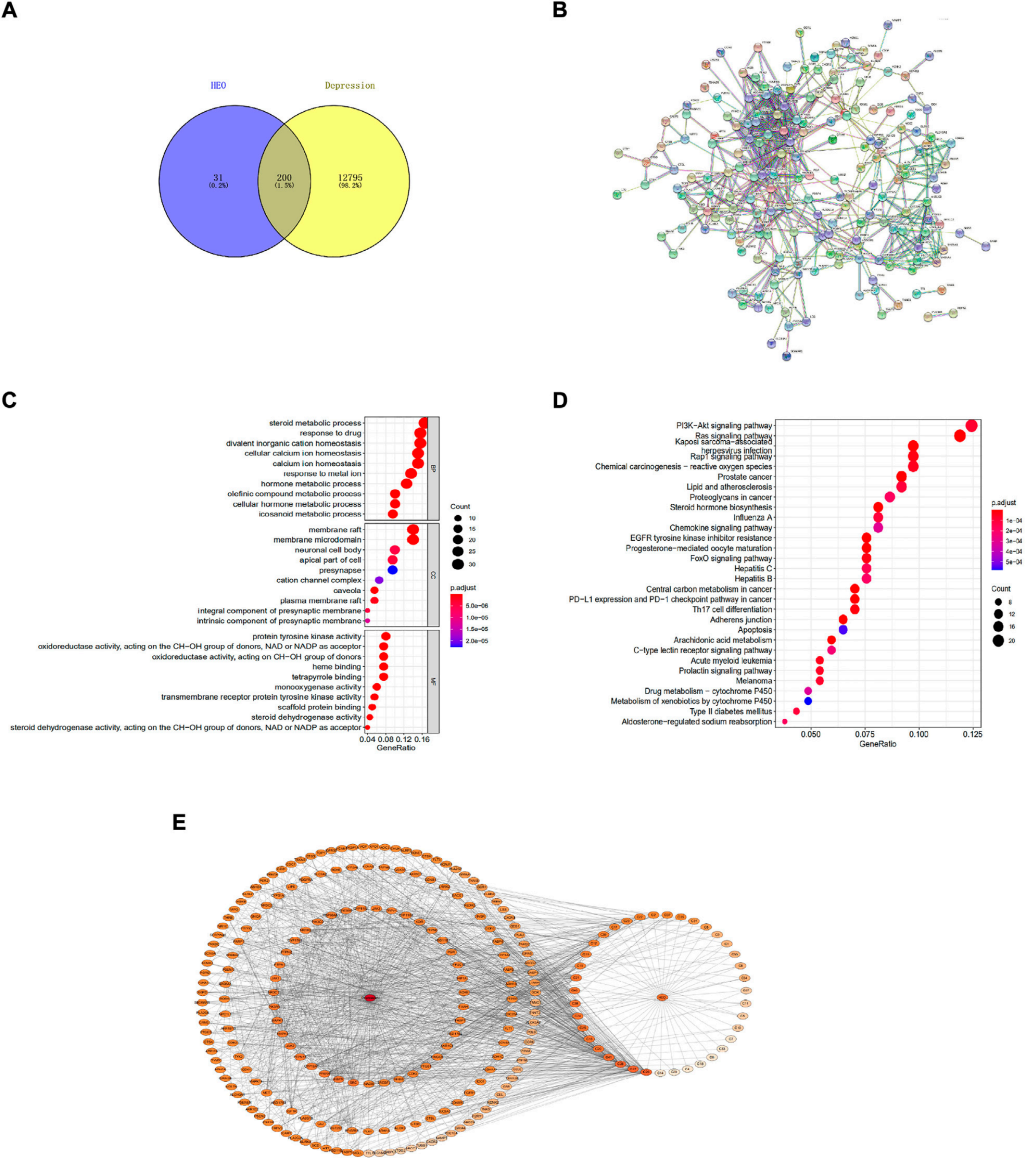
The network pharmacology method was adopted to predict potential mechanisms of HEO on depression. Venn diagram of depression-associated targets and predicted targets of active components **(A)**; Protein-protein interaction network (PPI) of target proteins **(B)**; Bubble chart of GO functions enrichment **(C)**; Bubble chart of KEGG pathways analysis of overlapping target genes (top 20) **(D)**; Drug-molecular-target-disease network diagram **(E)**. The color of nodes represents the degree’s value, and the deeper color represents a greater interaction degree.

As shown in Figure 7A, from the Swiss Target Prediction online database, we obtained 231 potential targets of HEO. The gene card database was used to collect 12,995 genes which related to depression. The Venn diagram then shows that 200 potential targets of active compounds were associated with depression after combining the target sets of active components and disease. Figure 7B shows that 200 proteins and 604 edges were obtained. The top 20 targets, which included PTPN11, JAK2, EGFR, JAK1, and TYK2, et al. Figure 7C displays the results of the GO functional enrichment analysis. In our network, BP mainly related to steroid metabolism, drug response, and cellular calcium ion homeostasis. The CC had membrane raft, membrane microdomain, and neuronal cell body et al. MF mainly comprised protein tyrosine kinase activity, et al. Then, KEGG pathways analysis was perform by the R language software, and bubble chart of that was shown in Figure 7D, mainly related to PI3K/Akt signaling pathway.

The drug-disease-target network diagram has 233 nodes and 1474 edges, including 39 molecules and 200 target proteins. The network diagram depicted the interaction between depression targets and active HEO components (Figure 7E).

### HEO treatment affected relative mRNA levels of PI3K/Akt and protein expression in the CUMS treatment mice

**FIGURE 8:**
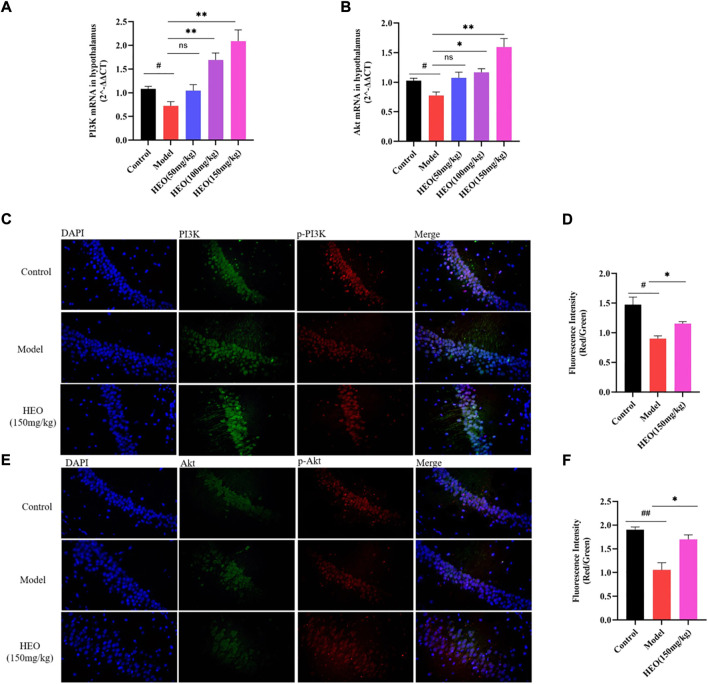
Effects of HEO on mRNA expressions of PI3K **(A)** and Akt **(B)**, and protein expression of PI3K/p-PI3K, Akt/p-Akt in hippocampus. Immunofluorescence images of PI3K (green), p-PI3K (red), and DAPI (blue) **(C)**; immunofluorescence images of Akt (green), p-Akt (red), and DAPI (blue), Merged images were shown in the right panel (400×) **(E)**; Quantification analysis of **(D)** and **(F)**. Data were expressed as mean ± SEM (*n* = 3). ^#^ *p* < 0.05, ^##^ *p* < 0.01 vs control group; *p < 0.05 vs the CUMS group.

The RT-qPCR results demonstrated that the model group’s relative mRNA levels of PI3K were decreased (Figure 8A, *p* < 0.05 vs control group). HEO treatments of 100 and 150 mg/kg increased expression levels (*p* < 0.01). Similarly, in the CUMS treatment group, relative mRNA expression of Akt was significantly decreased (Figure 8B, *p* < 0.05 vs control group). HEO treatment could increase the levels (*p* < 0.05). HEO treatment altered the relative mRNA levels of PI3K/Akt in CUMS-induced depression-like mice. We also studied the effect of HEO on the protein relative expression of PI3K, p-PI3K, Akt, and p-Akt. The results showed that the CUMS treatment group’s p-PI3K/PI3K and p-Akt/Akt ratios were significantly decreased (*p* < 0.05 and *p* < 0.01 vs control group, Figures 8C,D). After HEO (150 mg/kg) treatment, the ratio increased (*p* < 0.05 and *p* < 0.05 vs model group).

### HEO has protective effect on PC12 cells injured by CORT

**FIGURE 9:**
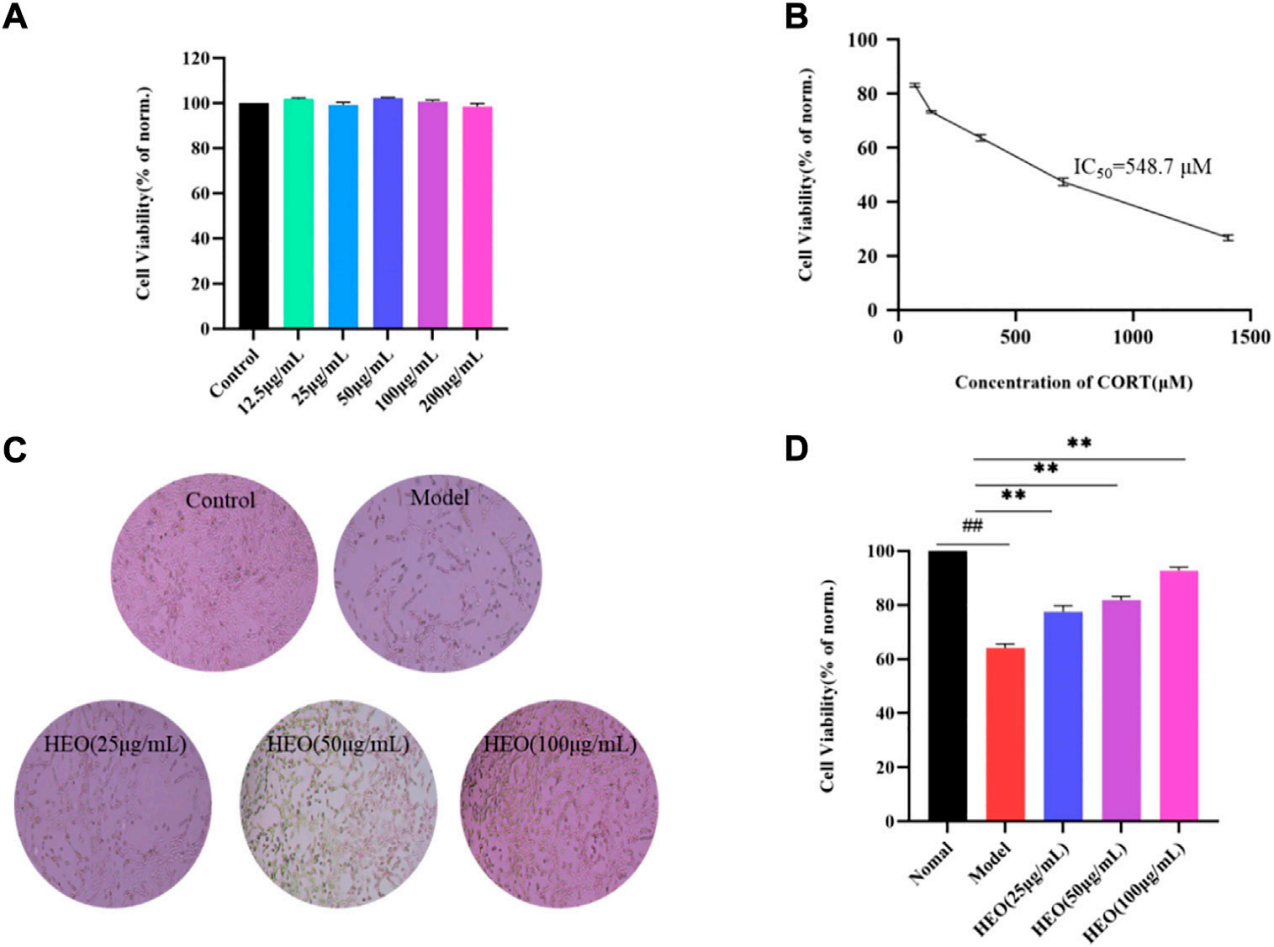
Protective effects of HEO on CORT-induced PC12 cells. Effects of HEO on the viability of normal PC12 cells **(A)**; Effects of various concentrations of CORT on the viability of PC12 cells **(B)**; The represented cell morphology of CORT-induced PC12 cells with HEO (×100) **(C)**. Effects of HEO (25 μg/ml, 50 μg/ml, and 100 μg/ml) on the viability of CORT-induced PC12 cells **(D)**. For data expressed as mean ± SEM (*n* = 3).^##^ *p* < 0.01 vs control group; **p < 0.01 vs model group.

Figure 9A depicts the effects of various HEO concentrations on the viability of PC12 cells after 4 h. HEO concentrations ranging from 12.5 to 200 μg/ml had no effect on PC12 cells when compared to the control group. Therefore, we chose the concentration of 25, 50, and 100 μg/ml of HEO as the low, medium and high concentration of HEO respectively. As shown in Figure 9B, the best working concentration of CORT with a working time of 24 h is 500 μM (IC50 = 548.7 μM). Furthermore, Figure 9D shows that HEO could improve the cell viability of CORT-stimulated PC12 cells (*p* < 0.01 vs model group). These results indicated that HEO potentially protects against inhibited CORT-treated PC12 cell injury.

### HEO suppresses apoptosis in CORT-stimulated PC12 cells

**FIGURE 10:**
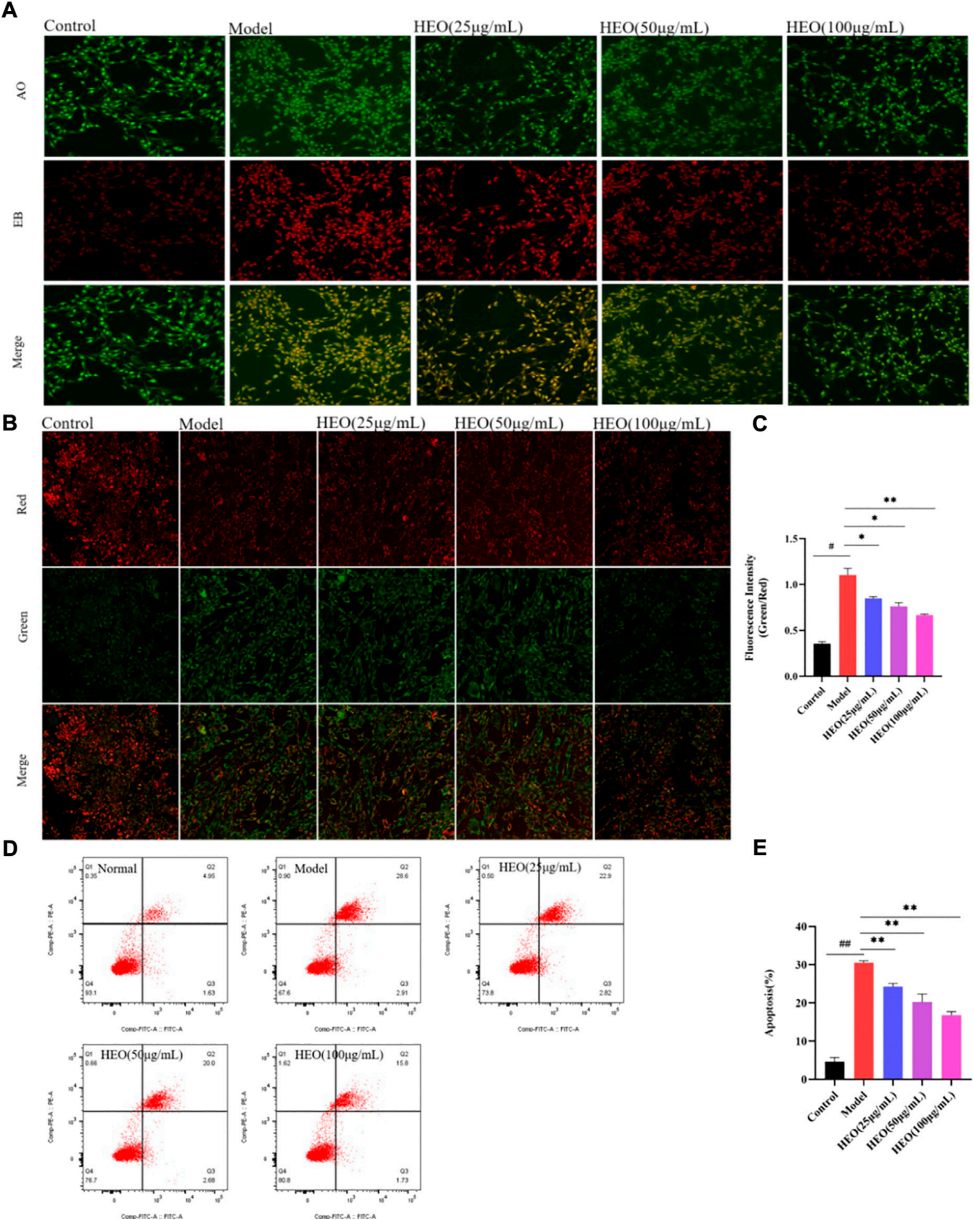
HEO suppresses apoptosis in CORT-treated PC12 cells. AO-EB staining (×20) **(A)**; Effects of HEO on the ΔΨm in PC12 cells (×40) **(B–C)**; Apoptotic assay by flow cytometry of PC12 cells **(D–E)**. The values were represented as the mean ± SEM (*n* = 3). ^#^ *p* < 0.05 and ^##^ *p* < 0.05 vs the control group; *p < 0.05 and **p < 0.01 vs the model group.

The HEO effective on apoptosis of PC12 cells stimulated by CORT was analyzed. In AO/EB staining, PC12 cells in the control group were almost uniformly green with uniform nuclei. However, the cells in the model group had irregular shapes and enhanced red fluorescence, indicating that PC12 cells were damaged by CORT induction. After administration of HEO, the red fluorescence was weakened and the typical morphology of green cells was significantly increased, indicating that apoptotic cells were significantly reduced (Figure 10A). In addition, we measured the green/red fluorescence ratio (MMP, Δψm), the effect of HEO on cell apoptosis was quantitatively studied. The increase of green/red fluorescence ratio was an early feature of cell apoptosis. Δψ was shown in Figures 10B,C. CORT significantly increased the green/red fluorescence ratio of PC12 cells (*p* < 0.01 vs control group). In contrast, HEO concentrations (25, 50, and 100 μg/ml) for 4 h could significantly reduce the green/red fluorescence ratio (*p* < 0.05, *p* < 0.05, and *p* < 0.01), indicating that HEO could reverse the decrease of MMP induced by CORT. In addition, after induction with 500 μM CORT for 24 h, the apoptosis rate of PC12 cells increased to 30.51%, while that of control cells was only 4.69% (*p* < 0.01). However, after 4 h of treatment with different concentrations of HEO (25, 50, and 100 μg/ml), the apoptosis rate decreased to 24.27% (*p* < 0.01), 20.27% (*p* < 0.01), and 16.82% (*p* < 0.01), respectively (Figures 10D,E). HEO could significantly reduce the apoptosis induced by CORT in PC12 cells.

### HEO regulates the phosphorylation of PI3K akt in CORT-treated PC12 cells

**FIGURE 11:**
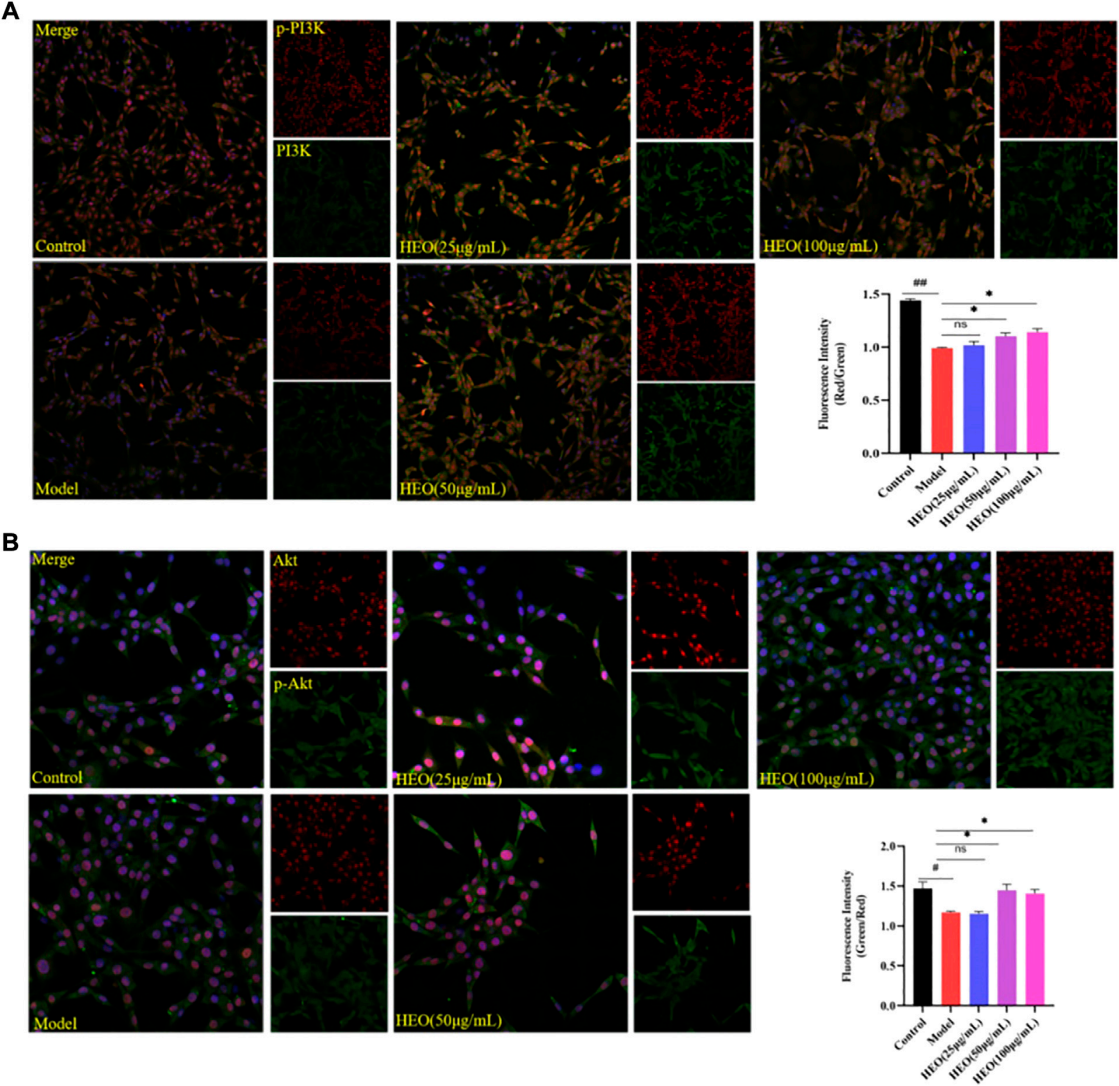
Effects of HEO on protein expressions of PI3K, p-PI3K, Akt, and p-Akt in CORT-treated PC12 cells. PC12 cells were pretreated with HEO (25, 50, and 100 μg/ml) for 4 h, and subsequently subjected to CORT (500 μM) for 24h. Protein expressions were determined by immunohistochemistry. p-PI3K(Red)/PI3K(Green) **(A)**; p-Akt (Green)/Akt (Red) **(B)**. The values were represented as the mean ± SEM (*n* = 3). ^##^ *p* < 0.01 and ^#^ *p* < 0.05 vs the control group; *p < 0.05 vs the model group.

The PI3K/Akt signaling pathway is important in the survival of neurons and the inhibition of apoptosis. Figures 11A,B shows that after 24 h of CORT treatment, the phosphorylation of PI3K in PC12 cells decreased significantly (*p* < 0.01 vs control group). The concentrations of 50 and 100 μg/ml HEO could significantly increase the phosphorylation of PI3K (*p* < 0.05, *p* < 0.05). A similar pattern was observed in the phosphorylation of Akt. Results above suggested that HEO may inhibit the apoptosis of PC12 cells induced by CORT via activating PI3K/Akt signaling pathway.

## Discussion

Aromatic essential oils are widely used to alleviate emotional diseases in the world (Sentari et al., 2019). The fruits of *Z. bungeanum* Maxim. is a dual-purpose product for medicine and food. According to research, *Z. bungeanum* Maxim. has a specific protective effect on the nervous system (Li R et al., 2020). The goal of this research was designed to investigate the HEO ameliorative effects on CUMS-stimulated depression-like mice and to speculate on its possible mechanism.

GC-MS was adopted to investigate the main antidepressant components of HEO. The results of the analysis revealed that the main components of HEO were linalool, d-limonene, and linalool acetate. Whether these components were the effective antidepressant components of essential oils needs further study.

The despair Behavior model could preliminarily evaluate the antidepressant activity of drugs (Czéh et al., 2016). In our study, the treatment of HEO (50, 100 and 150 mg/kg) could decrease immobility time in FST and TST. Results preliminary confirmation HEO has potential antidepressant activity. The occurrence of depression was related to many factors, so it was difficult for animal models to simulate the occurrence of depression completely. However, CUMS has been commonly applied to elucidate the pathological mechanism of depression and as a model to screen for the antidepressant potential of new drug molecules, so CUMS model is a better animal depression model accepted by researchers (Guo et al., 2019). In our present study, SPT, FST, and OFT were used to estimate whether the depression model was successfully made. Studies showed that patients with depression were often accompanied by anxiety (Hirschfeld, 2001). Therefore, the high price cross maze was (EPM) used to evaluate the anxiety state. The results of EPM showed that after HEO treatment, the time for animals to enter the open arm significantly increased, suggesting that HEO could alleviate anxiety, which was also reported in previous research (Wei et al., 2021). Administration with HEO ameliorate depression-like symptoms and increased the central area’s residence time and sucrose preference. In all, HEO manifested an antidepressant effect on the CUMS model mice.

Monoamines, such as 5-HT, DA, and NE, were the vital neuromodulators in the development of emotional disorders (Dhamija et al., 2017; Kraus et al., 2017). HEO could increase 5-HT, DA, and NE in the hippocampus, indicating that modulation of monoaminergic systems may be one of the antidepressant mechanisms of HEO. HPA axis is the regulatory hub of human neuroendocrine immune network composed of hypothalamus, pituitary, adrenal gland and downstream target organs, which plays a crucial role in maintaining homeostasis. HPA axis with hyperactivity was a common neurobiological abnormality in patients with depression. In particular, excessive secretion of corticotropin releasing hormone (CRH) and corticosterone (CORT) can mediate neurodegeneration and induce cognitive impairment and loss of mood in patients with depression. CUMS treatment significantly increased the level of CRH, then persistently stimulated the HPA axis, and ultimately the expression of CORT was increased (Fiksdal et al., 2018; Nedic Erjavec et al., 2021). HEO treatment could decrease levels of CRF and CORT, thus alleviating the CUMS-induced depression behavior in mice. Pathological sectioning of mouse brain tissue showed shrinkage and volume loss of hippocampal neurons in the model group, which was ameliorated by the drug administration. BDNF promotes neuron survival, growth, differentiation, and development and is involved in the structural and functional plasticity of neurons. BDNF’s synaptic plasticity can influence the release and transmission of DA, 5-HT, and adrenergic neurons, thereby influencing depression (Zhang et al., 2016; Björkholm and Monteggia, 2016). The results showed that after HEO and SERT administration, BDNF in hippocampus increased significantly compared to the model group.

Both DB model and CUMB model experiment results suggested that HEO possessed potential antidepression activity. However, the detailed molecular mechanisms remain unclear. Network pharmacology was considered as a useful to study traditional Chinese medicine (TCM) due to it can systematically analyze the interaction network of drug components, diseases, and protein targets. To predict potential mechanisms, we used the network pharmacology method. The compounds identified by GC-MS and KEGG analysis may be correlated to the PI3K/Akt pathway (Xiong et al., 2019; Zhang L et al., 2020). Furthermore, we used a CORT induced PC12 cell model to confirm the possible mechanism of HEO’s antidepressant activity. The cell model was the most commonly used model to investigate the potential mechanism of protective effects of some candidate drugs on neuronal cells (Zhang Q et al., 2020; Lin et al., 2021). Our present study exhibited that HEO possessed protective effects on PC12 cells induced by CORT. AO/EB double staining is an ideal method to evaluate the morphological changes of apoptotic nuclei. JC-1 was commonly considered a perfect probe for assessing ΔΨm. In addition, apoptosis rate was measure by flow cytometry analysis (Zhang et al., 2021a). Our results revealed that treatment with HEO remarkably decrease apoptosis in CORT-injured PC12 cells. The activation of PI3K/Akt signaling pathway is an important pathway for cell survival (Zhang et al., 2021b) and is also suggested to be therapeutically effective for depression via protection of neuron cells (Sun et al., 2021; Zhou et al., 2021). The mRNA of PI3K and Akt in hippocampus of model group mice was reduced, but after HEO treatment, the expression levels of the two genes significantly increased. However, in order to inhibit apoptosis, both the PI3K and Akt proteins must be phosphorylated. As a result, fluorescence double staining PI3K/p-PI3K and Akt/p-Akt were used to calculate the fluorescence intensity ratio of the two proteins. The experimental results confirmed that the phosphorylation of PI3K and Akt were increased after HEO treatment. Thus, HEO alleviated depression symptoms by activating PI3K/Akt signaling. However, more thorough work should be devoted to investigating the more in-depth mechanisms and possible substance basis of HEO for its effect.

## Conclusion

HEO significantly alleviated the depression-like behaviors of CUMS intervention mice with increased sucrose consumption and shortened immobile time. The antidepressant effect was might related to decrease of HPA axis hyperactivity, and increase the levels of neurotransmitters. Besides, HEO could improve the levels of BDNF, activate PI3K/Akt pathways to inhibit cell apoptosis. On these bases, we believe that HEO might be the potential active part of *Z. bungeanum* Maxim. For antidepressant.

## Data Availability

The original contributions presented in the study are included in the article/Supplementary Materials, further inquiries can be directed to the corresponding authors.
