# Supplementary material for: Anti-depression effectiveness of essential oil from the fruits of Zanthoxylum bungeanum maxim. on chronic unpredictable mild stress-induced depression behavior in mice
Source: Front Pharmacol. 2022 Sep 20;13:999962. doi: 10.3389/fphar.2022.999962 (PMC9530639; doi:10.3389/fphar.2022.999962)
Supplement: Supplementary file 1 [file Table1.docx]

**Table S1 Composition Analysis of the Essentional Oil from the fruits of**

***Zanthoxylum bungeanum*** **Maxim.**

| N | Identification | RT(min) | Molecular Mass | Structure | Relative Content (%) |
| --- | --- | --- | --- | --- | --- |
| 1 | (E,E)-2,4-Hexadienal | 5.944 | 96.13 | 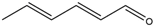 | 0.20 |
| 2 | α-Pinene | 6.557 | 136.23 | 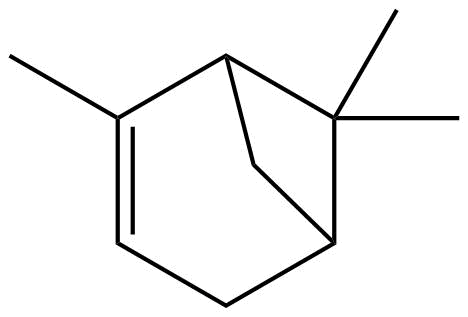 | 0.10 |
| 3 | 4-Thujene | 7.658 | 136.23 | 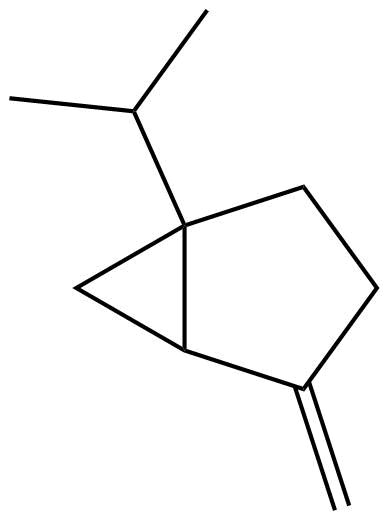 | 0.73 |
| 4 | β-Myrcene | 8.196 | 136.23 | 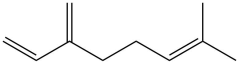 | 3.35 |
| 5 | 3-Thujene | 8.590 | 136.23 | 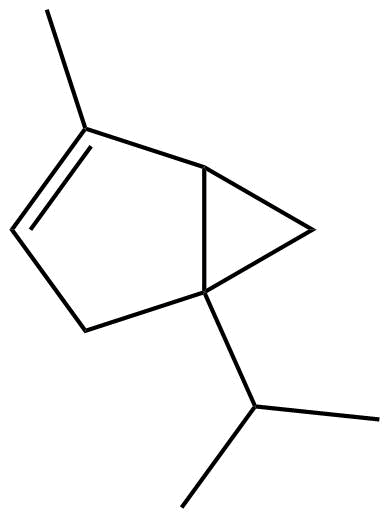 | 0.16 |
| 6 | Cyclohexene | 8.915 | 136.23 | 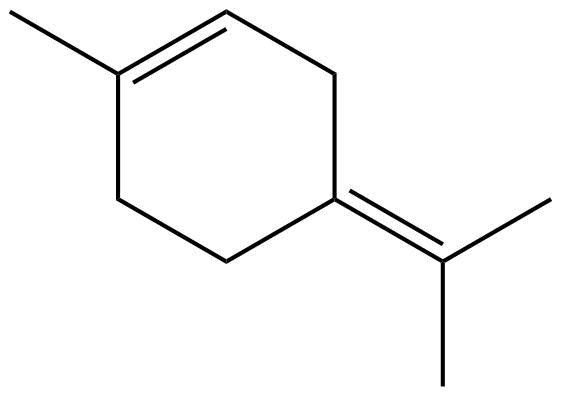 | 1.87 |
| 7 | o-Cymene | 9.159 | 134.21 | 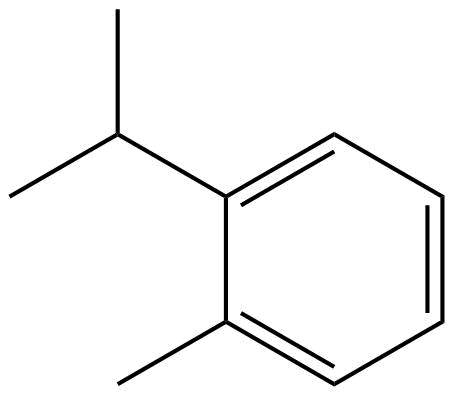 | 0.40 |
| 8 | D-Limonene | 9.359 | 136.23 | 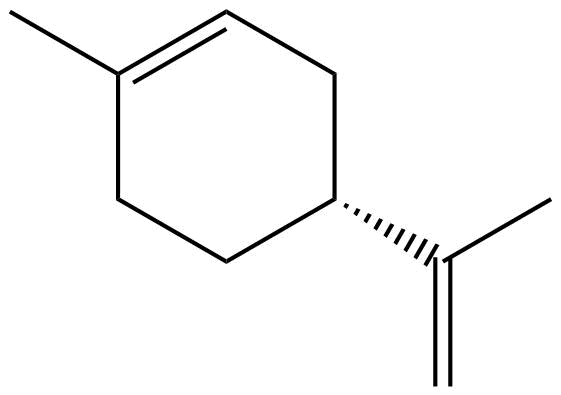 | 15.17 |
| 9 | Eucalyptol | 9.397 | 154.24 | 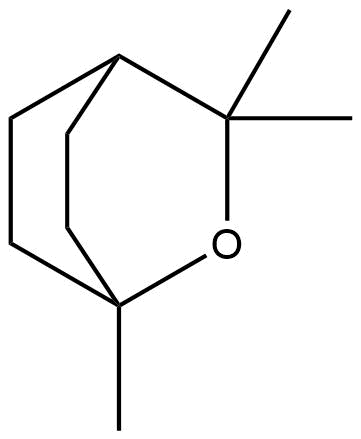 | 1.35 |
| 10 | trans-β-Ocimene | 9.574 | 136.23 | 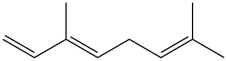 | 0.54 |
| 11 | β-Ocimene | 9.866 | 136.23 | 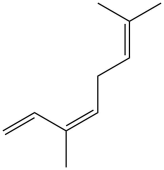 | 1.50 |
| 12 | γ-Terpinene | 10.179 | 136.23 | 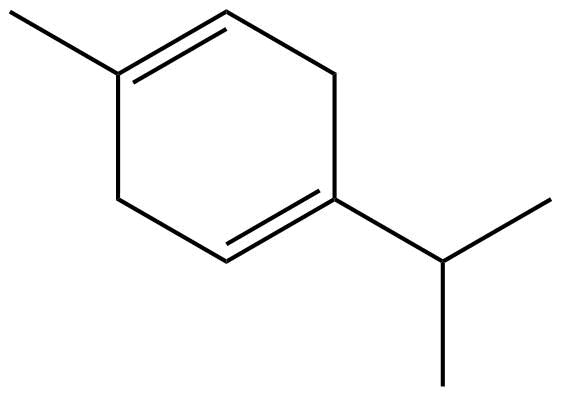 | 3.01 |
| 13 | [Linalool oxide B](https://www.ncbi.nlm.nih.gov/pcsubstance/?term=%22Linalool%20oxide%20B%22%5bCompleteSynonym%5d%20AND%206428573%5bStandardizedCID%5d) | 10.579 | 170.25 | 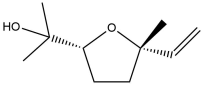 | 0.51 |
| 14 | Cyclohexene | 11.029 | 136.23 | 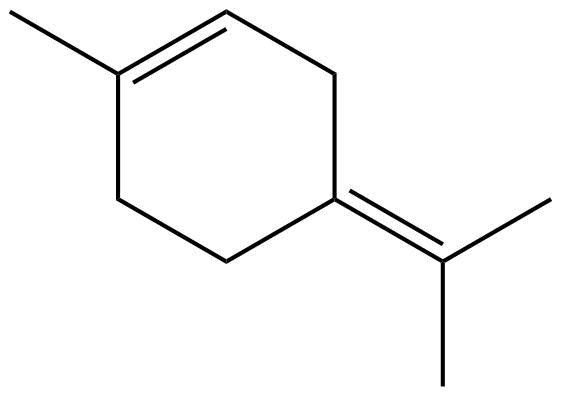 | 1.48 |
| 15 | Linalool | 11.624 | 154.25 | 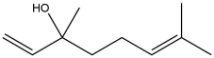 | 19.25 |
| 16 | 1,5,7-Octatrien-3-ol | 11.667 | 152.23 | 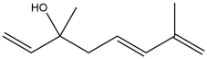 | 0.20 |
| 17 | 2-Cyclohexen-1-ol | 12.080 | 152.23 | 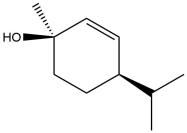 | 0.28 |
| 18 | 2,4,6-Octatriene, | 12.262 | 136.23 | 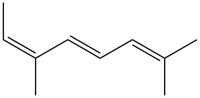 | 0.79 |
| 19 | 3-Cyclohexen-1-ol | 13.801 | 154.25 | 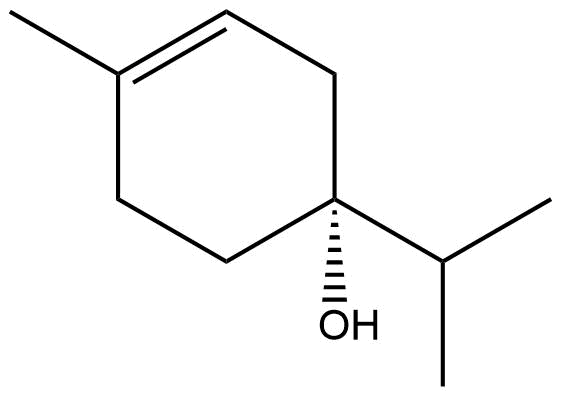 | 7.81 |
| 20 | 2-Cyclohexen-1-one | 13.969 | 138.20 | 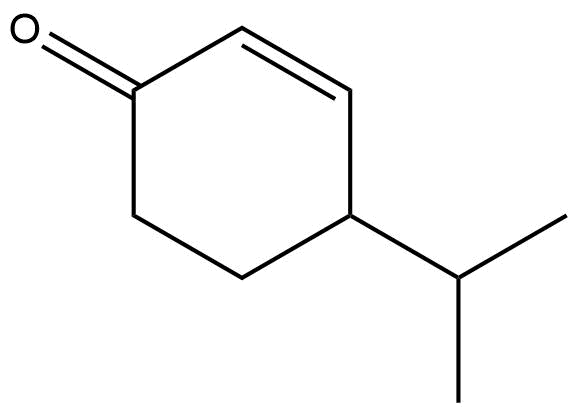 | 0.11 |
| 21 | L-alpha-Terpineol | 14.201 | 154.25 | 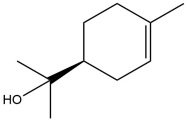 | 5.88 |
| 22 | 2-Cyclohexen-1-ol | 14.945 | 152.23 | 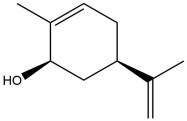 | 0.17 |
| 23 | Carveol | 15.289 | 152.23 | 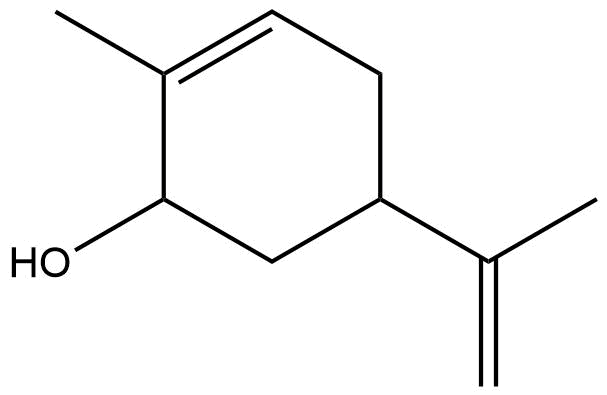 | 0.10 |
| 24 | D-Carvone | 15.615 | 150.21 | 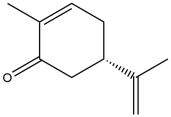 | 0.17 |
| 25 | Linalyl acetate | 16.027 | 196.29 | 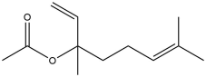 | 13.85 |
| 26 | α-Terpinyl acetate | 18.542 | 196.29 | 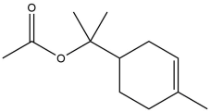 | 1.97 |
| 27 | 2,6-Octadien-1-ol | 18.942 | 196.28 | 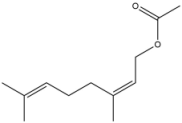 | 1.63 |
| 28 | Geranyl acetate | 19.487 | 196.29 | 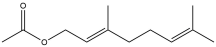 | 2.74 |
| 29 | Cyclohexane | 19.680 | 204.35 | 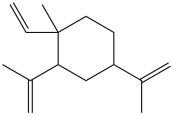 | 0.12 |
| 30 | β-Caryophyllene | 20.412 | 204.35 | 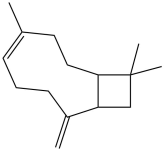 | 0.39 |
| 31 | γ-Elemene | 20.769 | 204.35 | 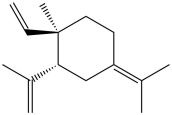 | 0.55 |
| 32 | Humulene | 21.301 | 204.35 | 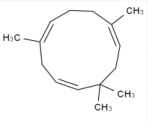 | 0.37 |
| 33 | Germacrene D | 22.014 | 204.35 | 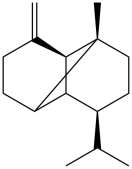 | 0.23 |
| 34 | α-Muurolene | 22.520 | 204.35 | 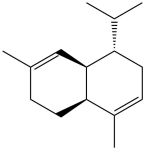 | 0.12 |
| 35 | Naphthalene | 22.902 | 164.28 | 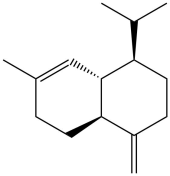 | 0.17 |
| 36 | Nerolidol | 24.466 | 222.37 | 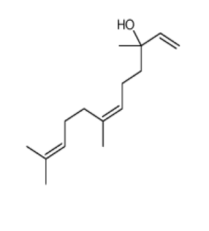 | 0.13 |
| 37 | 1H-Cycloprop[e]azulen-7-ol | 24.947 | 220.35 | 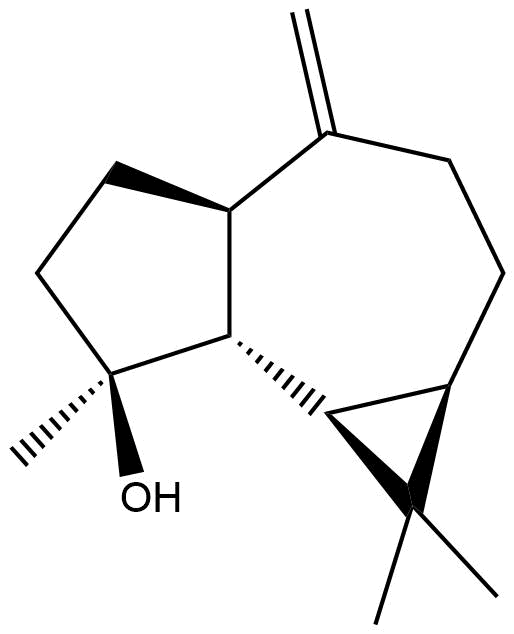 | 0.10 |
| 38 | tau-Muurolol | 26.680 | 222.37 | 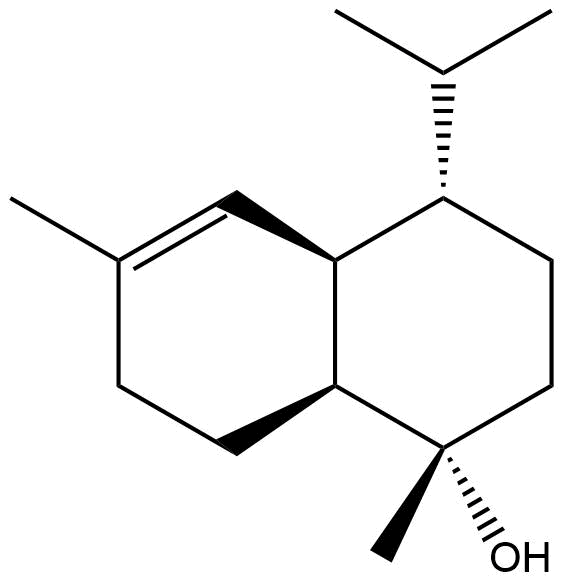 | 0.33 |
| 39 | 2-Naphthalenemethanol | 26.805 | 222.37 | 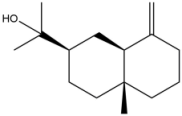 | 0.10 |
| 40 | α-Cadinol | 26.937 | 222.36 | 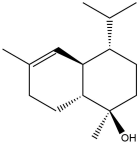 | 0.53 |
| 41 | α-Bisabolol | 27.537 | 222.36 | 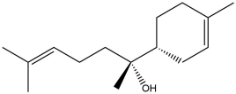 | 0.49 |
